# M60-like metalloprotease domain of the *Escherichia coli* YghJ protein forms amyloid fibrils

**DOI:** 10.1371/journal.pone.0191317

**Published:** 2018-01-30

**Authors:** Mikhail V. Belousov, Stanislav A. Bondarev, Anastasiia O. Kosolapova, Kirill S. Antonets, Anna I. Sulatskaya, Maksim I. Sulatsky, Galina A. Zhouravleva, Irina M. Kuznetsova, Konstantin K. Turoverov, Anton A. Nizhnikov

**Affiliations:** 1 Department of Genetics and Biotechnology, St. Petersburg State University, Universitetskaya nab., St. Petersburg, Russian Federation; 2 Laboratory of Amyloid Biology, St. Petersburg State University, Universitetskaya nab., St. Petersburg, Russian Federation; 3 Laboratory for Proteomics of Supra-Organismal Systems, All-Russia Research Institute for Agricultural Microbiology (ARRIAM), Podbelskogo sh., Pushkin, St. Petersburg, Russian Federation; 4 Institute of Cytology, Russian Academy of Science, St. Petersburg, Russian Federation; 5 Peter the Great St. Petersburg Polytechnic University, Polytechnicheskaya, St. Petersburg, Russian Federation; 6 Vavilov Institute of General Genetics, Russian Academy of Sciences, St Petersburg Branch, Universitetskaya nab., St. Petersburg, Russian Federation; University of Pittsburgh School of Medicine, UNITED STATES

## Abstract

Amyloids are protein fibrils with a characteristic spatial structure. Amyloids were long perceived as the pathogens involved in a set of lethal diseases in humans and animals. In recent decades, it has become clear that amyloids represent a quaternary protein structure that is not only pathological but also functionally important and is widely used by different organisms, ranging from archaea to animals, to implement diverse biological functions. The greatest biological variety of amyloids is found in prokaryotes, where they control the formation of biofilms and cell wall sheaths, facilitate the overcoming of surface tension, and regulate the metabolism of toxins. Several amyloid proteins were identified in the important model, biotechnological and pathogenic bacterium *Escherichia coli*. In previous studies, using a method for the proteomic screening and identification of amyloids, we identified 61 potentially amyloidogenic proteins in the proteome of *E*. *coli*. Among these proteins, YghJ was the most enriched with bioinformatically predicted amyloidogenic regions. YghJ is a lipoprotein with a zinc metalloprotease M60-like domain that is involved in mucin degradation in the intestine as well as in proinflammatory responses. In this study, we analyzed the amyloid properties of the YghJ M60-like domain and demonstrated that it forms amyloid-like fibrils *in vitro* and *in vivo*.

## Introduction

Amyloids are unbranched protein fibrils in which monomers form intermolecular β-sheets stabilized by numerous hydrogen bonds [1]. This structure, called cross-β, gives amyloids unusual resistance to treatment with ionic detergents (such as SDS and sarkosyl), leads to apple-green birefringence upon binding of with Congo red dye and enhances the fluorescence of Thioflavin-T dye [2,3]. Initially, amyloids were described as the lethal pathogenic agents of incurable diseases called amyloidoses in humans. To date, more than 30 pathological amyloids have been identified [4]. In recent decades, it has become clear that amyloids are not only involved in pathogenesis; they also play an essential role in a wide spectrum of biological processes occurring in different organisms, from archaea to humans. For example, in animals, functional amyloids control long-term memory [5,6], melanin polymerization [7], tooth enamel biomineralization [8], hormone storage [9], and programmed necrosis [10]. The greatest diversity of functional amyloids has been found in prokaryotes. In archaea, amyloids are involved in the formation of biofilms [11] and extracellular cell wall sheaths [12]. The main functions of bacterial amyloids are (i) biofilm formation (curlins, phenol-soluble modulins), (ii) overcoming surface tension (chaplins), and (iii) the metabolism of bacterial toxins (hairpins, microcins, listeriolysins) [13–15]. The roles of amyloids in the regulation of bacterial toxins are ambiguous: the amyloid state may be used for toxin storage (microcin E492) [16] or toxin inactivation (listeriolysin) [17], or amyloids may be functionally active (hairpin HpaG) [18].

*Escherichia coli* is one of the most prominent species of bacteria; its pathogenic strains are involved in different pathologies in humans and animals [19], while non-pathogenic strains are widely used in biotechnology and molecular genetics. Several amyloids have been identified in *E*. *coli* in recent years. First, P-type fimbriae composed of proteins called curlins have amyloid properties *in vivo*. The main structural and amyloid-forming protein of these fimbriae is CsgA curlin, while CsgB nucleates CsgA polymerization [20]. In addition, the membrane porins OmpA [21] and OmpC [22] form amyloid-like fibrils *in vitro*. Overall, only a few *E*. *coli* proteins have been tested for amyloid properties.

A proteomic method called PSIA (Proteomic Screening and Identification of Amyloids), which is based on the unusual resistance of amyloid fibrils to treatment with ionic detergents and allows the identification of candidates for amyloid-forming proteins in the complete proteomes of different organisms, was recently developed [23–25]. Using PSIA, we detected 61 potentially amyloidogenic proteins in the proteome of *E*. *coli* [24]. Almost all of these proteins contain potentially amyloidogenic regions predicted by the WALTZ bioinformatic algorithm [26], and 4 (BcsC, MukB, YfbK, and YghJ) have Q- and/or N-rich compositionally biased regions [27], which is a typical feature of many known amyloids [3,28,29]. YghJ is a secreted protein harboring an evolutionarily conserved zinc metalloprotease domain (aa 1081–1381) called M60-like [30]. According to recent observations, YghJ acts as a mucin-degrading metalloprotease involved in triggering proinflammatory responses [31] as well as in the colonization of the intestine [32] by pathogenic strains of *E*. *coli*. The M60-like domain was shown to be the main functional region of YghJ responsible for mucin degradation [32]. In this paper, we analyzed the propensity of the YghJ M60-like metalloprotease domain (YghJ_M_) to aggregate. We showed that YghJ_M_ formed unbranched fibrils that were resistant to treatment with ionic detergents and protease, had high content of β-sheets, bound Thioflavin-T and exhibited apple-green birefringence upon binding Congo red *in vitro*. Moreover, secreted YghJ_M_ formed amyloid-like fibrils at the surface of *E*. *coli* cells *in vivo*.

## Results

### M60-like metalloprotease domain of YghJ (YghJ_M_) forms detergent and protease-resistant aggregates with fibrillar morphology

**Fig 1:**
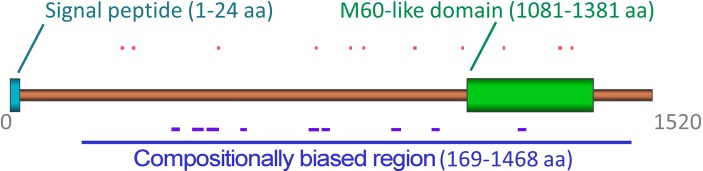
Bioinformatically predicted potentially amyloidogenic regions in the YghJ sequence. A schematic representation of the YghJ sequence is shown. The signal peptide (aa 1–24) and M60-like domain (aa 1081–1381) are indicated. Potentially amyloidogenic regions predicted by WALTZ [26] are colored in red, and potentially amyloidogenic regions predicted by ArchCandy [33] are colored in violet. The compositionally biased region predicted by SARP [27] is colored in dark blue.

The aminoacid sequence of YghJ is highly enriched with potentially amyloidogenic regions predicted by three different bioinformatic algorithms, WALTZ, SARP, and ArchCandy (Fig 1). These algorithms are completely different; WALTZ predicts short, potentially amyloidogenic regions based on a position-specific scoring matrix [26], SARP predicts compositionally biased regions [27], and ArchCandy detects potentially amyloidogenic β-arches [33]. The full-length YghJ bears 11 regions predicted by WALTZ (aa 261–268, 288–295, 489–495, 721–726, 802–807, 833–838, 953–960, 1067–1072, 1165–1170, 1296–1304, and 1325–1330), one large slightly asparagine-rich compositionally biased region predicted by SARP (aa 169–1468), as well as 9 potential β-arches predicted by ArchCandy [33] (aa 387–404, 435–460, 469–497, 551–566, 712–734, 742–760, 906–929, 1004–1021, and 1209–1226). The M60-like metalloprotease domain of YghJ, aa 1081–1381 (YghJ_M_), also has a high amyloidogenic propensity: it is compositionally biased and contains 3 amyloidogenic regions detected by WALTZ and one detected by ArchCandy (Fig 1).

**Fig 2:**
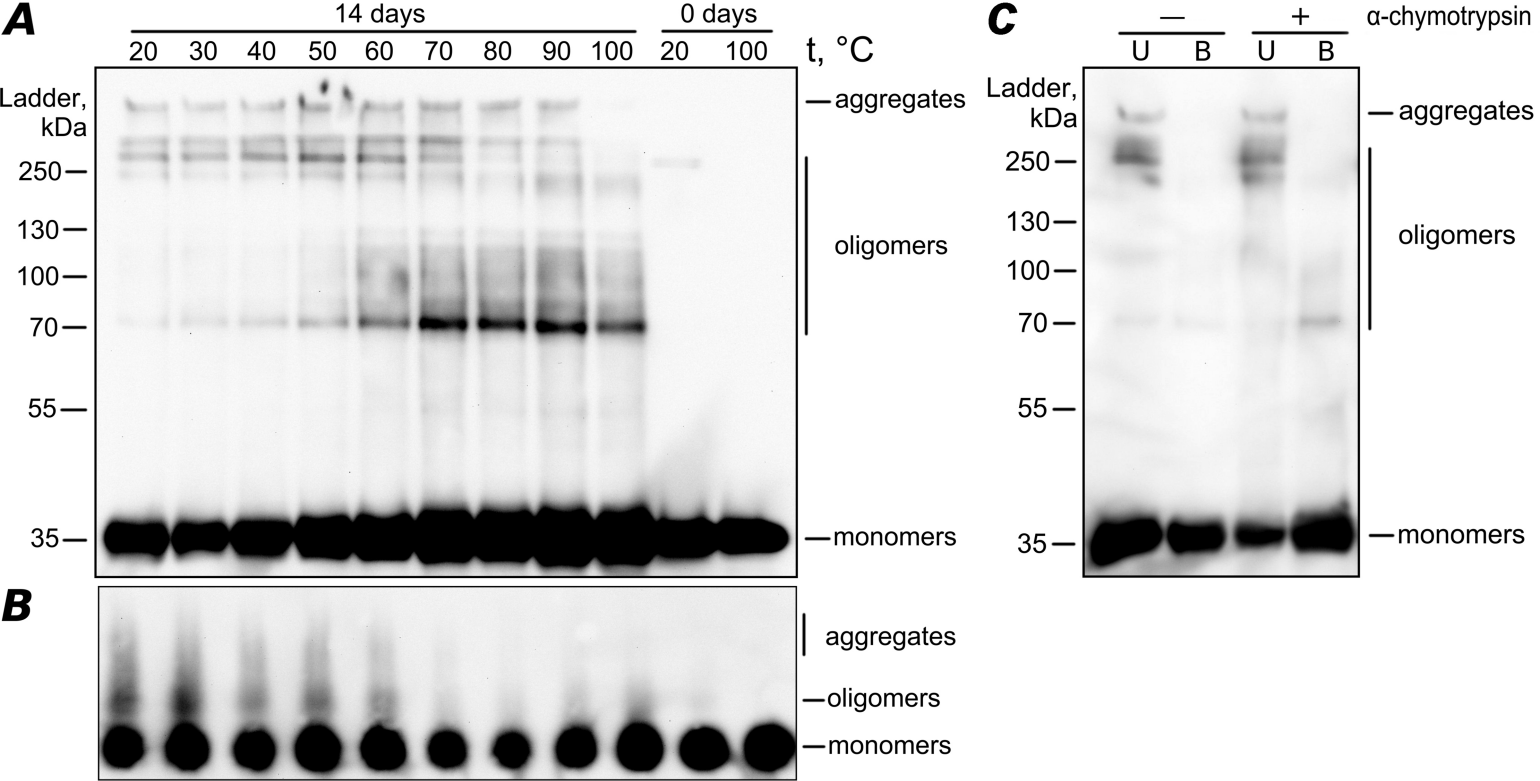
YghJ_M_ forms detergent- and protease-resistant aggregates. SDS-PAGE (A) and SDD-AGE (B) were used to visualize the YghJ_M_ detergent-resistant aggregates. In both cases, two YghJ_M_ samples were compared at the starting point (0 days) and after 2 weeks of incubation in non-denaturing conditions (14 days). The YghJ_M_ samples at the initial point of incubation were incubated with 2% SDS at 20°C or boiled at 100°C. The YghJ_M_ samples obtained at 14 days of incubation were incubated at temperature gradient from 20 to 100°C, as shown. The fractions of aggregates, oligomers, and monomers are indicated. A monoclonal anti-His_6_ antibody was used. The positions of protein weight ladder bands are shown (kDa). (C) The sample of the YghJ_M_ protein with concentration 1 mg/ml after 2 weeks of incubation in non-denaturing condition (see the “Experimental Procedures” section for details) was digested with α-chymotrypsin (Ct) at a 1:90 Ct-to-total protein mass ratio for 45 min at 20°C. After that reaction was terminated by addition of SDS-PAGE sample buffer. Then samples were immediately loaded onto the gel. Protein was detected by Western blotting with anti-His_6_ antibodies. “U”–unboiled samples, “B’–samples were additionally boiled for 5 minutes at 100°C prior the loading onto the gel. “-“–samples without Ct, “+”–samples treated with Ct.

To analyze the properties of YghJ_M_ aggregation, we used a plasmid encoding YghJ_M_ C-terminally tagged with His_6_ and under the control of an IPTG-inducible T7 promoter. YghJ_M_ was purified from *E*. *coli* and then transferred into non-denaturing conditions to allow spontaneous aggregation. At the initial point of incubation, no detergent-resistant aggregates of YghJ_M_ were detected, although a small portion of YghJ_M_ formed oligomers that were resistant to treatment with 2% SDS at 20°C (Fig 2A). After two weeks of incubation in the non-denaturing conditions (pH 7.4, 37°C), the picture of YghJ_M_ aggregation changed drastically (Fig 2). We detected detergent-resistant aggregates of YghJ_M_ by SDS-PAGE (Fig 2A) and SDD-AGE (Fig 2B). We found three distinct fractions of YghJ_M_: high-molecular-weight aggregates, oligomers, and monomers (Fig 2A and 2B). Aggregates analyzed with SDS-PAGE were stable in the presence of 2% SDS upon heating up to 90°C, while oligomers remained stable even after boiling at 100°C (Fig 2A). Notably, in the case of SDS-PAGE, the majority of the aggregates stayed in the wells without entering the gel (Fig 2A). Some of the oligomers passed through the stacking gel but stopped at the border of the resolving gel, and only a minor portion of the oligomers (most likely dimers) was observed at 70 kDa (Fig 2A). In the boiled samples, all the aggregates were destroyed, and only oligomers, mostly dimers, were found (Fig 2A). We observed a similar pattern on SDD-AGE, but in this case, the destruction dynamics of the aggregates were clearer as the temperature rose, and the most of the aggregates disappeared when the sample was heated at 70°C (Fig 2B).

Also, we analyzed the resistance of the YghJ_M_ aggregates to treatment with α-chymotrypsin protease (Ct) which is a component of pancreatic juice. The YghJ_M_ samples obtained after two weeks of incubation were treated with Ct at a 1:90 Ct-to-total protein mass ratio for 45 min at 20°C. After the incubation, samples were analyzed with SDS-PAGE. The Ct treatment resulted in the significant degradation of the YghJ_M_ monomers in the unboiled samples, while the amounts of the YghJ_M_ aggregates did not change (Fig 2C, lanes “U”–unboiled samples). Moreover, the number of monomers detected in the Ct-treated samples that were additionally boiled prior to loading onto the gel was significantly higher than in the untreated boiled samples. This observation confirms that YghJ_M_ aggregates are resistant to Ct at the indicated concentration (Fig 2C, lanes “B”–boiled samples). Taken together, YghJ_M_ forms detergent- and protease-resistant aggregates, which is a characteristic property of amyloids.

**Fig 3:**
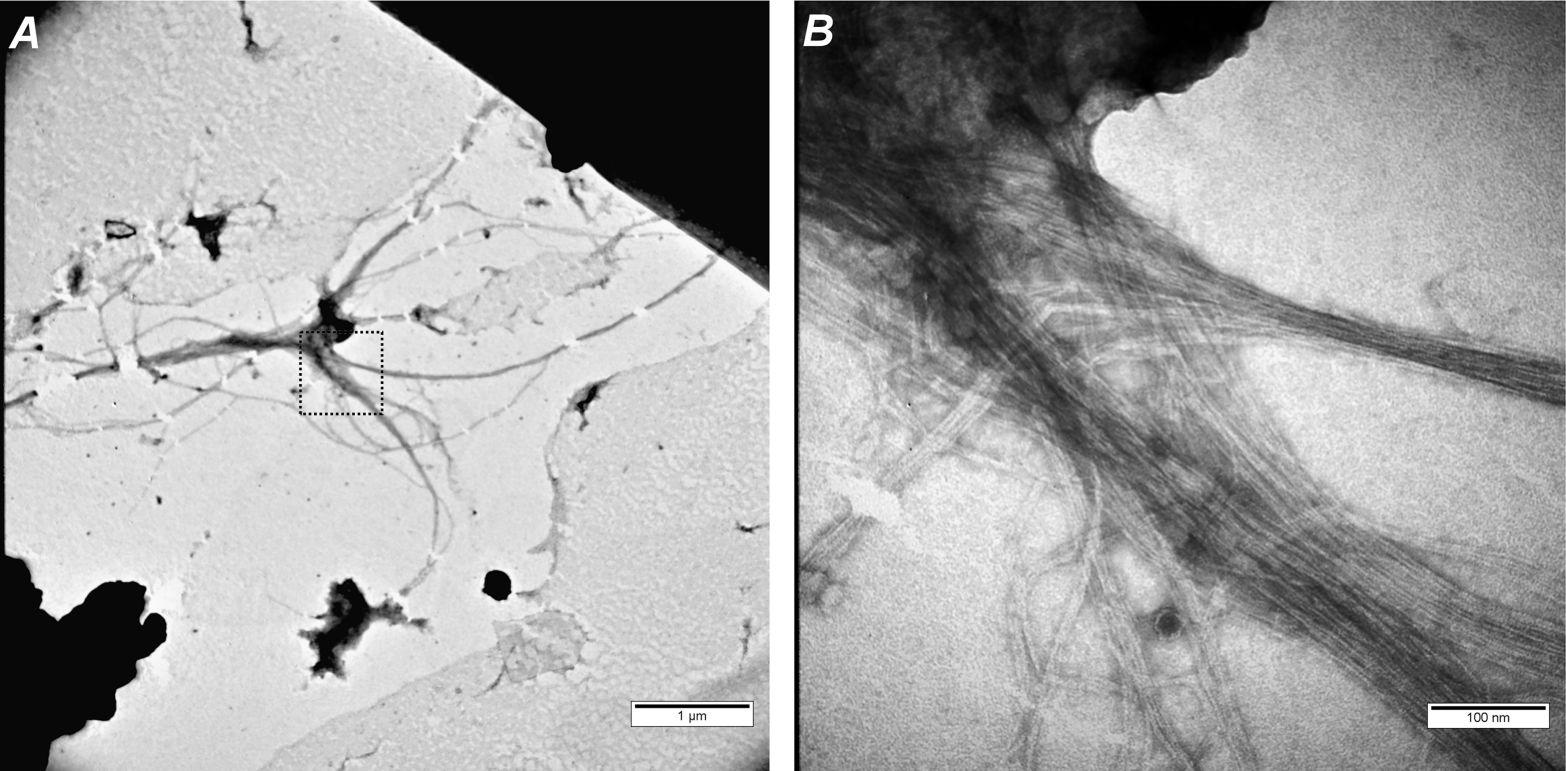
Aggregates of YghJ_M_ have fibrillary morphology. TEM images of YghJ_M_ fibrils after sonication and negative staining with uranyl acetate are shown. (A) Overall image at 20 000x magnification; (B) is a central dashed square from (A) at 200 000x magnification. Scale bars are indicated.

Next, we analyzed the morphology of the obtained YghJ_M_ aggregates using transmission electron microscopy (TEM). Initially, we found that YghJ_M_ formed very large aggregates that were difficult to analyze by TEM. To slightly break up these large structures, we processed the samples using ultrasonication. Then, we detected fibrils that were assembled in parallel into larger bundles (Fig 3). Notably, the average width of the fibrils composing the bundles was approximately 1.6 times smaller than that of the free fibrils (4.5 ± 0.7 nm vs. 7.5 ± 1.9 nm, respectively). This effect can be explained either by a more dense fit in the assembly of bundles or by a different interaction of free fibrils with the surface of the formvar film, on which the fibrils are somewhat flattened. The number of fibrils in the bundles varied but was clearly greater than ten. Accurate measurement of the fibril length was hampered due to its entanglement and high length which varies from 1.5 to 11 μm. This also did not allow estimating other characteristics of the fibrils, e.g. width or twist. The fibrils had elongated, predominantly unbranched morphology typical of amyloids (Fig 3).

### Fibril formation by YghJ_M_ leads to changes in its secondary structure

**Fig 4:**
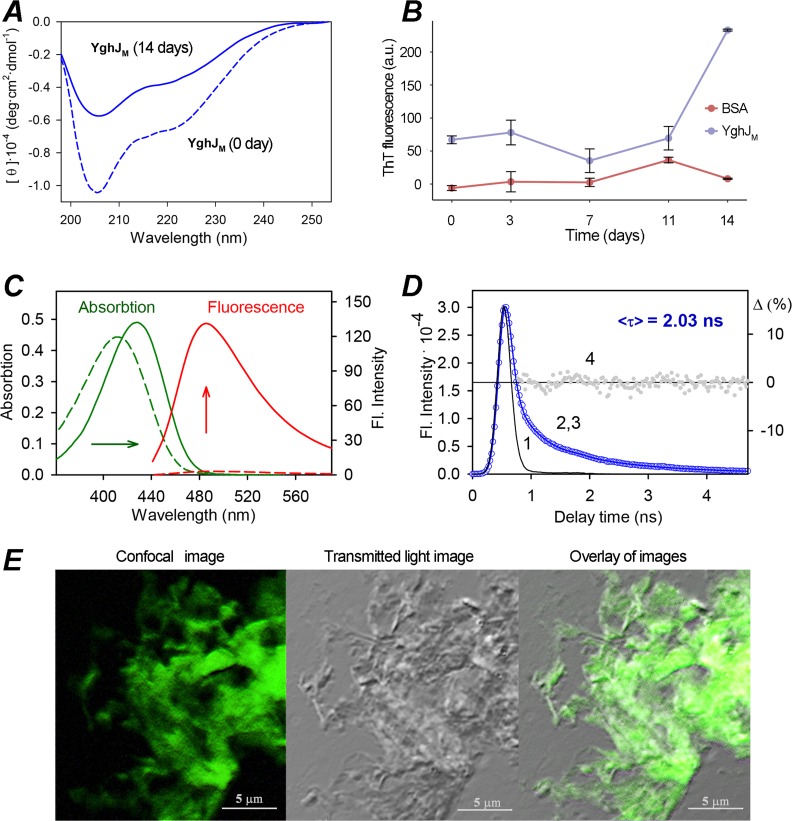
The analysis of Circular Dichroism and ThT binding of the YghJ_M_ fibrils. (A) Far-UV CD spectra of the YghJ_M_ samples at 0 days of incubation in non-denaturing conditions (dashed curve) and 14 days of incubation (solid curve) are shown. (B) The curves of the fluorescence spectra of ThT in the presence of BSA (negative control) and YghJ_M_ are shown. The time of the samples incubation in the non-denaturing conditions (days) is indicated. The means of ThT fluorescence (in arbitrary units, a.u.) are shown. (C) Detection of the YghJ_M_ fibrils with the use of ThT and photophysical properties of bound to fibrils dye. Absorption and fluorescence spectra of free ThT in buffer solution (dashed curves) and the dye in the presence of YghJ_M_ fibrils (solid curves) are shown. (D) Fluorescence decay curves of ThT bound to the YghJ_M_ fibrils. The excitation laser impulse profile (1), experimental decay curve of the bound dye fluorescence (2), best fit calculated fluorescence decay curve (3), and deviation between the experimental and calculated decay (4) are indicated. (E) Confocal microscopy image, transmitted light image and overlay of these images of the YghJ_M_ fibrils stained with ThT are shown.

The specific spatial structure of amyloids is characterized by the high content of β-strands [1]. Thus, in the case if YghJ_M_ forms amyloid fibrils, we might expect an increase in the content of β-sheets in the fibrillary YghJ_M_ (14 days of incubation) in comparison with the initial point of incubation of this protein. To investigate the changes in the secondary structure of YghJ_M_ resulting from the formation of the fibrils, CD spectra in the far-UV region were recorded (Fig 4A). The obtained results showed the differences in the forms of CD spectra of the YghJ_M_ at the initial point of incubation (0 days) and YghJ_M_ that formed fibrils (14 days) (Fig 4A). The decrease in the CD of fibrils is most likely due to their light scattering that, as it is known, distorts the CD spectra of macromolecules [34]. The analyses of the recorded spectra and the content of the secondary structure in the tested samples were carried out with the use of the different analysis programs and various sets of reference proteins (see Experimental Procedures). It was shown that the content of β-structure in the YghJ_M_ at the initial point of incubation was about 26% and α-helix was about 21%. The change in the secondary structure when YghJ_M_ forms the fibrils was observed: the amount of β-sheets increases (about 37%) and the amount of α-helices decreases (about 9%). These results are in a good agreement with the assumption that the β-structure plays an important role in the formation of amyloid fibrils that contain a cross-β spine with β-strands perpendicular to the fibril axis.

### The YghJ_M_ fibrils bind Thioflavin-T

Since YghJ_M_ formed detergent- and protease-resistant fibrils with the high content of β-sheets, we proposed that they might have amyloid properties. To test this hypothesis, we analyzed the effects of binding these fibrils with the amyloid-specific dyes Thioflavin-T (ThT) and Congo red. When ThT binds amyloid fibrils, the quantum yield of its fluorescence considerably increases [35]. We analyzed the ThT binding of YghJ_M_ samples during the fibril formation, from 0 to 14 days of incubation (Fig 4B). We found that the efficiency of ThT binding by YghJ_M_ significantly increases during the incubation of the sample (Fig 4B) indicating that the YghJ_M_ fibrils bind ThT.

Next, the interactions of ThT with the obtained YghJ_M_ fibrils, and, in particular, photophysical properties of dye bound to fibrils were investigated (Fig 4C). It was shown that the absorption spectrum of ThT in the presence of YghJ_M_ fibrils is significantly red-shifted (more than 15 nanometers) in comparison to that of free ThT in buffer solution (Fig 4C). This can be explained by the orientational dipole–dipole interaction of the free dye molecules with a polar solvent and more hydrophobic microenvironment of the dye bound to fibrils. A slight increase in ThT absorption at the maximum of the spectrum (which means the molar extinction coefficient increase) was also observed, that may be due to change in the dye molecules conformation upon their incorporation into fibrils.

The observed shift of the ThT fluorescence spectrum (a few nanometers) is less pronounced than the shift of the dye absorption spectrum. This may be due to the fact that the fluorescence of ThT in the solutions is caused by the transition from a nonequilibrium excited state to the nonequilibrium ground state, and the position of the peak of fluorescence spectrum is not so sensitive to the solvent polarity as the position of the maximum of the absorption spectrum. At the same time, the fluorescence intensity of the bound to YghJ_M_ fibrils dye increases in about 30 times in comparison to free dye in a buffer solution (Fig 4C). The fluorescence intensity of free ThT molecules is low because its benzothiazole and aminobenzene rings can rotate relative to one another in the excited state (that is typical for the class of molecular rotors to which ThT belongs), and the transition of the excited dye molecules to a state with a close to 90° angle between its fragments leads to nonradiative transitions to the ground state. A significant increase in the fluorescence intensity when the dye is incorporated into amyloid fibrils is caused by a restriction of the rotational motion of ThT fragments relative to each other in the excited state [36]. The significant increase of ThT fluorescence intensity in its binding to YghJ_M_ fibrils was also demonstrated with the use of confocal microscopy (Fig 4E).

We also recorded the fluorescence decay curves of ThT bound to YghJ_M_ amyloid fibrils (Fig 4D) and determined that the fluorescence lifetime of the bound to fibrils dye is about 2 ns. In the recent works, it was experimentally determined that the fluorescence lifetime of free ThT in aqueous solutions is about 1 ps [37]. In aqueous solutions, the rate of radiation-less deactivation of the excited state of ThT molecules process is much higher than the rotation of ThT molecule as a whole, and the fluorescence lifetime is determined by the rate of internal rotation of benzothiazole and aminobenzene rings related to each other. The Increase of the ThT fluorescence lifetime about 3 orders of magnitude when it binds to the YghJ_M_ fibrils (Fig 4D) in comparison with the free dye in buffer solution can be caused by the restriction of this rotation and decrease of the rate of the radiation-less deactivation of the dye molecules. Taking together, our results show that YghJ_M_ fibrils are bound specifically with ThT (Fig 4B and 4E) and exhibit typical for other amyloids photophysical properties (absorption spectra red-shift and significantly increased intensity and lifetime of the dye fluorescence, Fig 4C and 4D) suggesting their amyloid properties.

### The YghJ_M_ fibrils exhibit apple-green birefringence upon binding with Congo red

**Fig 5:**
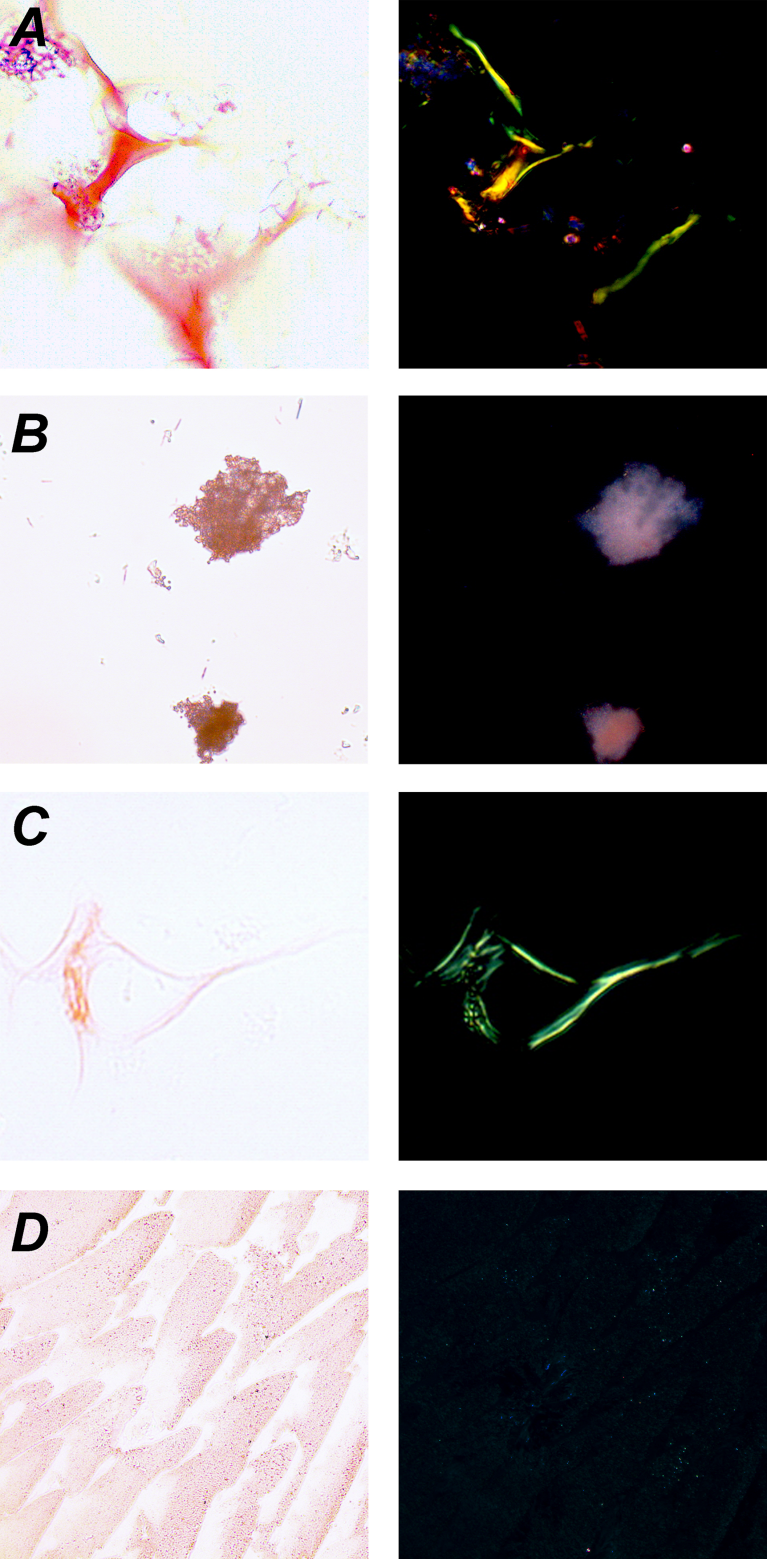
YghJ_M_ fibrils show birefringence upon binding Congo red. Protein samples obtained *in vitro* were stained with Congo red as described in “Experimental procedures” and examined under a transmitted light microscope fitted with crossed polarizers. (A) Sup35NM fibrils (positive control); (B) YghJ_M_ sample, 0 days of incubation; (C) YghJ_M_ fibrils, 14 days of incubation; (D) BSA (negative control). Left column–transmitted light; right column–polarized light. An objective with 20x magnification was used.

Next, we analyzed the Congo red staining of YghJ_M_ samples. Binding of Congo red azo dye with amyloids results in apple-green birefringence under polarized light [38], and this assay is considered the “gold standard” proof of the amyloid state of protein fibrils [39]. We used Congo red to stain YghJ_M_ samples obtained at 0 and 14 days of incubation and analyzed the effects under a polarizing microscope. The YghJ_M_ protein at the initial point of incubation formed large conglomerates that did not show apple-green birefringence (Fig 5B). After 14 days of incubation, YghJ_M_ fibrils exhibited clear apple-green birefringence (Fig 5C). These data corresponded to the results of ThT staining. The effects of staining of YghJ_M_ fibrils with Congo red (Fig 5B) were similar to those observed for the fibrils of the control amyloid protein, Sup35NM (Fig 5A), whereas the negative control, BSA, did not exhibit apple-green birefringence (Fig 5D), similar to the YghJ_M_ sample analyzed at the initial point of incubation (Fig 5B). Taken together, an array of experiments demonstrated that *in vitro*, YghJ_M_ fibrils possess the key features of amyloids: they are resistant to the treatment with ionic detergents and protease, have high content of β-sheets, increase the fluorescence of ThT, and exhibit apple-green birefringence upon Congo red binding.

### YghJ_M_ forms amyloid-like fibrils *in vivo*

**Fig 6:**
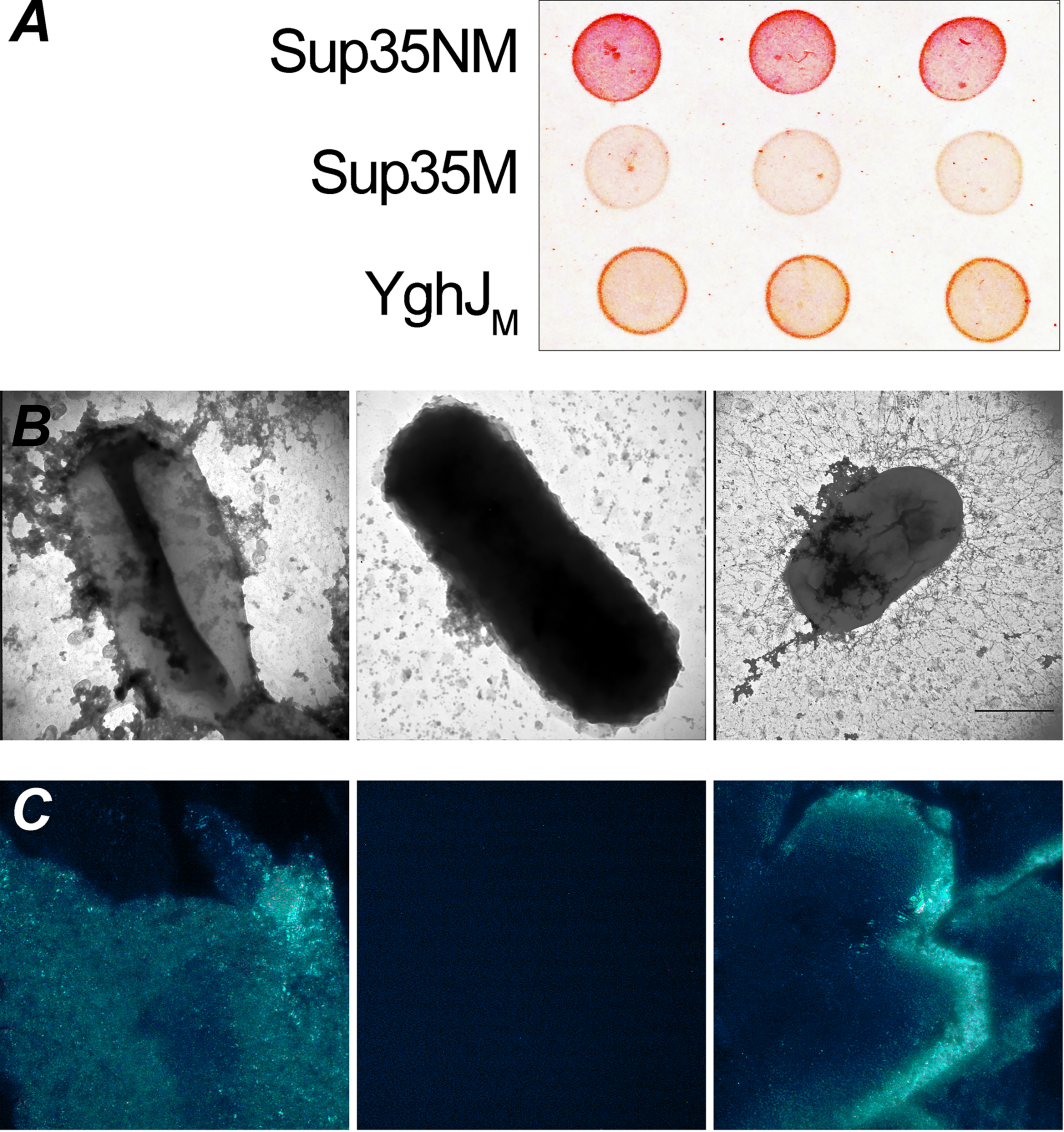
YghJ_M_ forms amyloid-like fibrils *in vivo*. (A) Phenotypic effects of YghJ_M_, Sup35NM, and Sup35M surface export by the C-DAG system in *E*. *coli* (for a detailed description, see “Experimental procedures”). An LB plate with 10 μg/ml Congo red is shown. The picture was taken after 5 days of incubation at 22°C. (B) (Left to right) TEM images of *E*. *coli* exporting Sup35NM, Sup35M, and YghJ_M_. (C) Cross-polarization study of the effects of Sup35NM, Sup35M, and YghJ_M_ surface export in *E*. *coli* cells. Cells were taken from the Congo red-containing plates after 5 days of incubation at 22°C. Cells were air-dried and analyzed under polarized light. An objective with 40x magnification was used.

To test whether YghJ_M_ forms amyloid-like fibrils *in vivo*, we used the curli-dependent amyloid generator system (C-DAG) for the export of the target protein to the surface of bacterial cells, where the fibril formation can be monitored *in vivo* either with electron microscopy or with Congo red staining. We used C-DAG plasmids facilitating the export of the yeast amyloid Sup35NM protein as the positive control and Sup35M, which cannot form fibrils, as the negative control. First, we tested the phenotypes of the bacterial cells exporting YghJ_M_, Sup35NM, or Sup35M on plates containing Congo red. Bacterial cells that export Sup35NM are red on such media, indicating the presence of fibrils at the cell surface, while Sup35M-expressing cells are white or pale since they do not secrete amyloid fibrils (Fig 6A). The cells exporting YghJ_M_ had a bright orange color, suggesting that this protein formed fibrils (Fig 6A).

We analyzed the cells expressing YghJ_M_, Sup35NM, and Sup35M using TEM. As expected, cells expressing Sup35NM were surrounded by a filamentous extracellular network, while the cells expressing Sup35M had a smooth surface (Fig 6B). The cells exporting YghJ_M_ formed numerous long, thin fibrils (Fig 6B). We also analyzed whether YghJ_M_ fibrils that formed *in vivo* exhibited birefringence. For this experiment, we took bacterial cells from the Congo red plates and analyzed them under a transmitted light microscope fitted with crossed polarizers. As expected, cells exporting YghJ_M_ and Sup35NM showed birefringence, while the cells exporting Sup35M did not exhibit this feature (Fig 6C). Taken together, we demonstrated that YghJ_M_ was able to form amyloid-like fibrils at the surface of bacterial cells *in vivo*.

## Discussion

YghJ plays an important role in the virulence of enterotoxigenic strains of *E*. *coli*; these strains are among the most important causes of infectious diarrhea, which presumably results in increased infant mortality in developing countries [40]. YghJ is a highly conserved protein [41,42], and it was recently demonstrated to be secreted by 89% of enterotoxigenic *E*. *coli* isolates [43] and specifically bind and degrade intestinal mucins [32]. Moreover, YghJ was required for the efficient delivery of the heat-labile toxin of *E*. *coli* to intestinal epithelial cells [32], triggered the induction of proinflammatory cytokines *in vitro* [31], and caused tissue damage in the mouse ileum [44].

Previously, we found that when expressed at endogenous levels, full-length YghJ was present in the fraction of the aggregates resistant to treatment with ionic detergents (SDS and sarkosyl) [24]. The YghJ amino acid sequence has unique properties since it is extremely rich in potentially amyloidogenic regions predicted by different bioinformatic algorithms (Fig 1). Considering these data, we proposed that aggregates of this protein might have amyloid properties. In this study, we demonstrated that the M60-like metalloprotease domain of the YghJ protein is able to form amyloid fibrils *in vitro* and *in vivo*. Although the kinetics of YghJ_M_ *in vitro* were relatively slow (we detected detergent-resistant fibrils after 14 days of sample incubation), the conditions of fibril formation were closer to physiological conditions, i.e., 37°C and pH = 7.4. Moreover, when we expressed YghJ_M_ in the C-DAG system [45] *in vivo*, *E*. *coli* cells formed numerous long extracellular fibrils and exhibited characteristic apple-green birefringence (Fig 6). Considering that full-length YghJ was present in the fraction of detergent-resistant aggregates and that its metalloprotease domain formed amyloid fibrils *in vitro* and *in vivo*, we suggest that YghJ might be a naturally occurring amyloid-forming protein in *E*. *coli*. The amyloid formation by YghJ might accumulate its molecules and decelerate their degradation in aggressive environment of the intestine. The amyloids are extremely stable, and such effect of the fibril formation seems very possible. In addition, various bacterial toxins and virulence factors undergo limited proteolysis before activation [46,47]. The involvement of the metalloprotease domain of YghJ in the core structure of the amyloid fibrils could potentially regulate the efficiency of its limited proteolysis by either bacterial or host proteases followed by functional activation.

Several *E*. *coli* proteins associated with the virulence of this bacterium have amyloid properties. Extracellular curli fibers composed of several proteins, the main structural subunit of which is CsgA [48], have amyloid properties [20]. These extracellular *E*. *coli* amyloids were shown to be associated with urinary-source bloodstream infections [49]. Two outer membrane proteins, the porins OmpA and OmpC, form amyloid fibrils [21,22]. Additionally, similar to YghJ, these proteins were detected in the fraction of *E*. *coli* proteins resistant to treatment with ionic detergents [24]. Bacterial outer membrane proteins are known to be involved in neurological diseases and can cross the blood-brain barrier [50]. While OmpA was demonstrated to form amyloid fibrils *in vitro* [21], the amyloid properties of OmpC were shown in protein extracted from cells [22]. Moreover, the intramuscular injection of OmpC led to spongiform neurodegeneration in a mouse model [22]. Thus, *E*. *coli* amyloid-forming proteins are likely to play important roles in the pathogenesis of this bacterium. Our data on the amyloid properties of the YghJ M60-like metalloprotease domain contribute to the knowledge on bacterial amyloids and could be useful to investigations of the relationship between amyloidogenesis and *E*. *coli* virulence.

## Experimental procedures

### Plasmids

To express the YghJ M60-like metalloprotease domain, i.e., aa 1301–1381 (YghJ_M_), C-terminally fused with a His_6_ tag, an Alicator kit (Thermo Scientific, USA) was used. The YghJ_M_-encoding fragment was PCR-amplified using the primer pair YghJ1081CCF and YghJ1381CCR (S1 Table) and genomic DNA from *E*. *coli* strain DH5α [51] as the template. The PCR-amplified fragments were inserted into the pAlicator vector according to the manufacturer’s recommendations. The correctness of the resulting pAc-YghJ_M_ plasmid was verified by sequencing with the primers provided by the manufacturer (Thermo Scientific, USA).

To construct the plasmid for YghJ_M_ export to the *E*. *coli* cell surface, the pExport vector [45,52] was used. A sequence encoding the YghJ_M_ fragment flanked by *Not*1 and *Xba*1 restriction sites was PCR-amplified using primers YghJ1081Not1F and YghJ1381XbaIR and the pAc-YghJ_M_ plasmid as the template. The resulting PCR product was inserted into the pExport vector linearized at the *Not*1 and *Xba*1 restriction sites. Other plasmids for C-DAG expression containing sequences encoding yeast Sup35NM (aa 2–253) and Sup35M (aa 125–253) were constructed previously [45,52].

### Expression, purification, and fibrillation of proteins

For protein purification, *E*. *coli* strain BL21 was used. The overproduction of recombinant proteins was carried out in 2TYa media supplemented with 0.1 mM IPTG. Cultures were grown at 37°C for 4 h. Proteins were purified in denaturing conditions (in the presence of 8 M urea) according to a previously published protocol [53] without the Q-sepharose purification step. A one-step purification procedure with a Ni-NTA agarose (Invitrogen) column was performed. Proteins were concentrated using a Centricon (30 kDa, Millipore). To obtain aggregates of YghJ_M_, the proteins were diluted at least 100-fold in fibril assembly buffer (5 mM potassium phosphate, pH 7.4, containing 150 mM NaCl) to a final protein concentration of 0.5 mg/mL. Samples were incubated at 37°C with slow end-over-end rotation (Bio RS-24 rotator, Biosan) for 14 days. Under these non-denaturing conditions, YghJ_M_ spontaneously aggregates. For several experiments, fibrils were sonicated with a Bandelin sonoplus device (Teopal) for 2 seconds at 57% device power (3 cycles).

### Immunochemical analysis

Protein extraction, SDS-PAGE, semi-dry transfer onto PVDF membranes (Amersham), and western blot hybridization were performed according to standard protocols [54]. The protocol for SDD-AGE is described in [55]. Capillary transfer was performed as described [56]. Before loading on the SDS-PAGE and SDD-AGE gels, the samples were incubated at different temperatures (as indicated in the text) for 5 minutes.

For the experiments with trypsin digestion, the samples of the YghJ_M_ protein (1 mg/ml) after 2 weeks of incubation in non-denaturing condition were treated with α-chymotrypsin (Ct) (Sigma-Aldrich) at a 1:90 Ct-to-total protein mass ratio for 45 min at 20°C. Then the reaction was terminated by addition of SDS-PAGE sample buffer and samples were immediately loaded onto the gel.

A mouse monoclonal anti-His antibody (#27-4710-01 GE Healthcare) was used to detect YghJ_M_ fused with a His_6_ tag. An anti-mouse secondary antibody was also used to detect the anti-His antibody. Proteins were visualized using ECL Select Western Blotting Detection Reagent (Amersham), and GeneGnome hardware and software (Syngene) was used for imaging.

### Electron microscopy

For TEM measurements, formvar-coated copper grids were used in conjunction with negative staining with a 1% aqueous solution of uranyl acetate. A Jeol JEM-2100 (Japan) transmission electron microscope was used. Samples of *in vitro-*generated YghJ_M_ fibrils were sonicated 3 times for approximately 2 seconds at a power of 57% on a Bandelin sonoplus (Teopal) device.

### Congo red binding assay for *in vitro*-generated fibrils

To perform the Congo red assay for protein samples obtained *in vitro*, a saturating amount of NaCl was added to 80% ethanol and filtered. Then, a saturating amount of Congo red (Sigma-Aldrich) was added to the solution and filtered again. Protein samples (5–10 μl) were placed on a microscope slide and air-dried. Next, 100 μl of Congo red solution was added and air-dried again. Finally, an excess of Congo red was removed by washing the slide with 96% ethanol. Apple-green birefringence was analyzed under a DMR XA transmitted light microscope (Leica) fitted with crossed polarizers and equipped with 20x and 40x dry objectives.

### Thioflavin-T binding assay

ThT was purchased from Sigma-Aldrich. Fluorescence measurements were performed using a Cary Eclipse spectrofluorometer (Agilent Technologies) according to the manufacturer’s recommendations at 0, 3, 7, 11, and 14 days of incubation of the protein samples in non-denaturing conditions. Fluorescence spectra were obtained using 445 nm light for excitation and an emission wavelength of 485 nm. The protein concentration was 0.5 mg/mL, and the dye was added to a final concentration of 12 μM.

### Spectral measurements

The samples of "UltraPure Grade" ThT from AnaSpec were used without further purification. ThT was dissolved in 2 mM Tris-HCl buffer (pH 7.7) with 150 mM NaCl.

The absorption spectra were recorded using a U-3900H spectrophotometer (Hitachi). For these experiments, the microcells (5 × 5 mm, cell type 101.016-QS) were used (Hellma). The concentration of ThT in solutions was determined using a molar extinction coefficient of *ε*_*412*_ = 3.16·10^4^ M^-1^cm^-1^ (according to the results of our measurements).

Fluorescent measurements were performed using Cary Eclipse spectrofluorometer (Agilent Technologies) with FLR cells (10 × 10 × 4 mm, cell type 26.400-F) with a path length of 10 mm (Starna Scientific). The fluorescence intensity in experiments was excited at the wavelength *λ*_*ex*_ = 430 nm. The recorded values for fluorescence intensity were corrected for the primary inner filter effect (for a detailed description, see [57]).

Circular dichroism (CD) spectra were obtained using a Jasco-810 spectropolarimeter (Jasco). The far-UV CD spectra were recorded in a 1 mm path length cell from 260 to 197 nm, with a step size of 0.2 nm. An average of three scans was obtained for all spectra. The CD spectra of the buffer solution were recorded and subtracted from the protein and fibrils spectra. CD spectra analysis was performed with the use of CDPro software package that consists of three programs for determining the secondary structure content (SELCON3, CDSSTR and CONTIN). This software has nine sets of reference proteins constructed from the reference sets generally used in CD analysis. Analysis that was carried out by the use of the different programs and various sets of reference proteins for the each tested sample gave the similar results and the content of the secondary structure was estimated as an average of the determined values.

### Time-resolved fluorescence measurements

The fluorescence decay curves were recorded by a spectrometer FluoTime 300 (Pico Quant) with the Laser Diode Head LDH-C-440 (*λ*_*ex*_ = 440 nm). For performing the experiments the microcells (5 × 5 mm, cell type 101.016-QS) were used (Hellma). The measured emission decays were fit to a multiexponential function using the standard convolute-and-compare nonlinear least-squares procedure [58]. In this method, the convolution of the model exponential function with the instrument response function (IRF) was compared to the experimental data until a satisfactory fit was obtained. The IRF was measured using cross correlation of the excitation and fundamental gate pulse. The fitting routine was based on the nonlinear least-squares method. Minimization was performed according to Marquardt method [59].

### Confocal microscopy

The confocal laser scanning microscope Olympus FV 3000 (Olympus) with an objective lens with a 60x magnification and an aperture NA 1.42 was used to obtain the images of the ThT-stained fibrillar structures of the YghJ_M_ protein. The fixed excitation laser line 405 nm was used. Registration of ThT fluorescence was carried out in the range of 420–520 nm. To assess the presence of fibrils in the investigated sample region, the transmitted light images were also obtained.

### C-DAG experiments

To export the YghJ_M_, Sup35NM, and Sup35M proteins, *E*. *coli* strain VS39 was transformed with the corresponding pExport-based plasmids (see “Plasmids” section). A detailed description of the C-DAG protocol has been published in [45]. Briefly, transformants were selected on LB media supplemented with 100 μg/ml carbenicillin, 25 μg/ml chloramphenicol, and 0.5% glucose. Single colonies were grown overnight in 3 ml of liquid LB with 100 μg/ml carbenicillin and 25 μg/ml chloramphenicol at 37°C with agitation. For the Congo red color assay, a dilution of an overnight culture (OD 0.01) was incubated in 3 ml of liquid LB with 100 μg/ml carbenicillin, 25 μg/ml chloramphenicol for 30 min at 37°C. Then, 5 μl of the cultures were spotted onto the “Congo red inducing” LB plates (with 100 μg/ml carbenicillin, 25 μg/ml chloramphenicol, 0.2% L-arabinose, 1 mM IPTG, 10 μg/ml Congo red), as well as the corresponding control plates [45], and grown for 5 days at 22°C. To analyze the apple-green birefringence, cells were taken from the plate, resuspended in phosphate-buffered saline, placed onto a microscope slide, air-dried, and analyzed with a DMR XA transmitted light microscope (Leica) fitted with crossed polarizers and equipped with a 40x objective. For TEM experiments, cells were grown in the same conditions on plates without Congo red [45].

### Bioinformatic analysis

The analyses of protein sequence for the presence of potentially amyloidogenic regions were performed using three different algorithms, WALTZ [26], SARP [27], and ArchCandy [33]. The following settings were used: high-specificity mode and pH 7.0 for WALTZ; probability threshold of 10^−8^ for SARP; and 0.576 threshold for ArchCandy.

## Supporting information

S1 Table Oligonucleotides used in this study. (PDF)

## Abbreviations

BSA: Bovine Serum Albumin
CD: Circular Dichroism
Ct: α-chymotrypsin
IPTG: Isopropyl β-D-1-thiogalactopyranoside
SDD-AGE: Semi-denaturing Detergent Agarose Gel Electrophoresis
PSIA: Proteomic Screening and Identification of Amyloids
SDS: Sodium Dodecyl Sulfate
SDS-PAGE: Sodium Dodecyl Sulfate Polyacrylamide Gel Electrophoresis
Sarkosyl: Sodium Lauroyl Sarcosinate
SARP: Sequence Analysis based on the Ranking of Probabilities
TEM: Transmission Electron Microscopy
ThT: Thioflavin-T
