# Supplementary material for: M60-like metalloprotease domain of the Escherichia coli YghJ protein forms amyloid fibrils
Source: PLoS One. 2018 Jan 30;13(1):e0191317. doi: 10.1371/journal.pone.0191317 (PMC5790219; doi:10.1371/journal.pone.0191317)
Supplement: S1 Table — (PDF) [file pone.0191317.s001.pdf]

S1 Table. Oligonucleotides used in this study

Oligonucleotide	Sequence
YghJ1081CCF	AGAAGGAGATATAACTATGGGTAACATGCAGTCAACTGGC
YghJ1381CCR	GTGGTGGTGATGGTGATGGCCCTCTGCCCATTCCTTCAGCT
YghJ1081Not1F	TAGTAGCGGCCGCAGGTAACATGCAGTCAACTGGC
YghJ1381XbaIR	TGTTCTCTAGATTACTCTGCCCATTCCTTCAGCT
